# Extract of a Letter from Heidelberg

**Published:** 1837-07

**Authors:** 


EXTRACT OF A LETTER FROM HEIDELBERG, DATED MAY 23, 1837.

...... On my way here, I visited Brussels, Liége, and Bonn.... I did not see much of eye-diseases in the Military Hospital of Brussels; but in that of Liége I saw a very great number of cases.... I was introduced to Dr. Vlemincke, inspector-general of the Belgian army medical department, who gave me a general order of admission for all the military hospitals in Belgium; he, moreover, gave me two books, which I will here notice. The first as to date is entitled "Rapport à M. le Ministre Directeur de la Guerre, Baron Evain, sur l'Ophthalmie de l'Armée; precédé de Considerations générales sur l'Etiologie de cette Affection; par J. F. Vlemincke, M.D." Dr. V. endeavours to show that the ophthalmia of the army is not propagated so much by contagion as by other more appreciable and palpable causes; among the principal of which he particularly insists on the peculiar dress of the soldiers,—the tightness of the collars of their coats, and weight of their caps; and this he infers from the circumstance that none but soldiers have been affected, and that the ophthalmia has not prevailed in the French army, the soldiers' clothes not being made so as to produce any undue compression of the neck or head; whilst, in the Prussian army, where the contrary is the case, the ophthalmia has also prevailed. He, moreover, mentions that it was the infantry among whom the disease principally raged; and these, he tells us, are the very men who have the most inconvenient clothing, and who are the longest time on duty in full dress. In consequence of the representations of Dr. V., the fashion of the soldiers' clothes has now been brought to resemble more that of the French, and it appears that the ophthalmia has been on the decline since. To affirm, with Dr. V., that this is entirely owing to the change of dress, would perhaps be going rather too far; but I have no doubt that the removal of the compression of the neck is a powerful prophylactic. Anxious to diffuse among the medical officers of the army as correct a knowledge of the pathology of this scourge as possible, Dr. V. has got translated, for their use, Dr. Eble's very excellent monography on the Structure and Diseases of the Conjunctiva. The title of the French translation is "De la Structure et des Maladies de la Conjunctive, avec des Considérations particulières sur l'Ophthalmic contagieuse, par Burchard Eble, M.D., Médecin de Regiment au Service d'Autriche, &c." There is prefixed to the translation an introduction by Dr. V., in which he very much recommends the study of ophthalmology, and defends his own opinions as to the cause of the ophthalmia of the army, from the attacks of his numerous opponents, the contagionists.

In Belgium, there are four universities; those of Louvain, Gand, Liége, and the "Université libre" of Brussels. The latter was established about three years ago, by subscription: a medical school had existed before. The power of conferring degrees has been taken away from the universities. Degrees are now granted by a jury of examinators, appointed by the government, the senate, and house of representatives. The jury sits in Brussels, and thither every candidate for a degree must come to be examined, no matter where he has studied. The candidate for the degree of M. D. must first obtain a degree in science. No particular course of study is required, no particular period of study, nor age: all that is necessary is knowledge sufficient to enable the candidate to undergo the examination. The jury is very strict in their examination; so that there will be fewer graduates in Belgium now than formerly.

The "Université libre" of Brussels is governed by a council chosen by the subscribers. The council appoint the professors. There are ordinary and honorary professors, and *aggregés.* There are about three hundred students altogether, but of these very few are medical. In regard to the duration of the courses, Dr. Guiette told me that his course of Physiology lasts the whole year; three lectures a week. The fees of the university are,—*Droit d'inscription*, 15 francs; *Retribution annuel*, 200 francs.

---

### BOOKS RECEIVED FOR REVIEW.

